# Baclofen alters gustatory discrimination capabilities and induces a conditioned taste aversion (CTA)

**DOI:** 10.1186/1756-0500-4-527

**Published:** 2011-12-09

**Authors:** Gina N Wilson, Orion R Biesan, Jennifer L Remus, G Andrew Mickley

**Affiliations:** 1 Neuroscience Program and Department of Psychology, Baldwin-Wallace College, Berea, OH 44017, USA

## Abstract

**Background:**

Studies intending to measure drug-induced changes in learning and memory are challenged to parse out the effects of drugs on sensory, motor, and associative systems in the brain. In the context of conditioned taste aversion (CTA), drugs that alter the sensorium of subjects and affect their ability to taste and/or feel malaise may limit the ability of investigators to make conclusions about associative effects of these substances. Since the GABAergic system is implicated in inhibition, the authors were hopeful to use the GABA agonist, baclofen (BAC), to enhance extinction of a CTA, but first a preliminary evaluation of BAC's peripheral effects on animals' sensorium had to be completed due to a lack of published literature in this area.

**Findings:**

Our first experiment aimed to evaluate the extent to which the GABA_B _agonist, BAC, altered the ability of rats to differentiate between 0.3% and 0.6% saccharin (SAC) in a two bottle preference test. Here we report that 2 or 3 mg/kg (i.p.) BAC, but not 1 mg/kg BAC, impaired animals' gustatory discrimination abilities in this task. Furthermore, when SAC consumption was preceded by 2 or 3 mg/kg (i.p.) BAC, rats depressed their subsequent SAC drinking.

A second experiment evaluated if the suppression of SAC and water drinking (revealed in Experiment 1) was mediated by amnesiac effects of BAC or whether BAC possessed US properties in the context of the CTA paradigm. The time necessary to reach an asymptotic level of CTA extinction was not significantly different in those animals that received the 3 mg/kg dose of BAC compared to more conventionally SAC + lithium chloride (LiCl, 81 mg/kg) conditioned animals.

**Conclusions:**

Our findings were not consistent with a simple amnesia-of-neophobia explanation. Instead, results indicated that 2 and 3 mg/kg (i.p.) BAC were capable of inducing a CTA, which was extinguishable via repeated presentations of SAC only. Our data indicate that, depending on the dose, BAC can alter SAC taste discrimination and act as a potent US in the context of a CTA paradigm.

## Background

GABAergic (γ-Aminobutyric acid) systems play an important role in learning, memory, and inhibitory processes [1-3]. Baclofen [BAC; (±)-β-(Aminomethyl)-4- chlorobenzenepropanoic acid.] is a GABA_B _receptor agonist that has been used extensively in studies aimed at determining the role of GABA receptors in learning and memory. Baclofen causes passive avoidance deficits [4], spatial memory impairments [1,5,6] impairments in memory retention [3] and other disturbances of memory either through the disruption of acquisition and/or consolidation of learned responses [3,4,6,7]. Additionally, GABA_B _antagonists have been shown to attenuate both BAC-induced and scopolamine-induced spatial working and reference memory deficits in the Morris water maze [8].

A variety of behavioral and physiological side effects linked to GABAergic system manipulations have obscured our understanding of the role this system may play in memory formation and retention. For example, hypodipsia, sedation, vertigo [9], and hyperphagia [10] have followed BAC administration. Rats injected intraperitoneally with BAC displayed both hyperdipsic and hyperphagic responses [10,11]. These behaviors were reversed, in rats, using systemic and intracerebroventricular injections of the GABA_B _antagonist, CGP35348 [11]. Infusions of BAC in the median raphe nuclei also led to increased eating behaviors, but did not affect drinking behaviors [12].

Other laboratories failed to find BAC-induced changes in appetitive behaviors. For example, Chester and Cunningham [13] showed that BAC does not alter ethanol-induced conditioned taste aversion in mice, nor does BAC, itself, have malaise inducing properties at a dose of 2 mg/kg. Experiments by Jacobson and colleagues [4,14] used genetically altered mice lacking specific subtypes of the GABA_B _receptor and reported a vital role for the GABA_B _system in conditioned emotional responses and conditioned taste aversion (CTA) acquisition/extinction. For example, it was demonstrated that GABA_B(1a) _knockout mice fail to acquire a CTA while GABA_B(1b) _knockouts failed to extinguish a CTA [14].

Given the role of the GABA_B _system in mediating learning and memory and its specific involvement in CTA learning and consummatory behaviors, combined with the increasing frequency of BAC use in learning, memory and addiction research, we were drawn to investigate the effects of BAC on acquisition and extinction using the CTA paradigm in rats. The aforementioned side-effects of BAC have not been extensively investigated in the context of how they may adversely influence the interpretation of taste-dependant learning paradigms, specifically the CTA paradigm. A CTA may be acquired when an animal consumes a novel taste (conditioned stimulus; CS) and then experiences symptoms of poisoning (unconditioned stimulus; US). When later given a choice between the poisoned taste and water, the animal will avoid the taste previously associated with malaise [15]. However, when designing animal experiments that utilize the CTA methodology along with systemic administration of neuro-active drugs, there are several considerations to which an investigator must attend: (a) does the drug alter basal food or liquid consumption? (b) does the drug compromise the animals' ability to perceive the conditioned stimulus (CS)? (c) does the drug cause malaise itself, thus acting as an US or interfering with actions of an US? Without this information, investigators may draw erroneous conclusions, attributing a drug-induced disruption in performance to a drug-induced learning deficit rather than a drug-induced alteration in sensory capabilities and concurrent task performance. Due to the disparity of findings regarding BAC and other GABA_B _system agonists' effects on consummatory behaviors [4,10,11,14], the present experiments attempted to establish the feasibility of using BAC in behavioral studies that employ taste paradigms, specifically CTA. Specifically, the aims of the present studies were to (a) determine whether or not BAC, at doses of 1, 2, or 3 mg/kg (i.p.) altered the perceived gustatory discrimination capability of animals in a two bottle preference test of SAC (0.3% SAC versus 0.6% SAC), and (b) to determine, via a CTA paradigm, if the observed deficiencies in gustatory discrimination capabilities may have been due to possible US effects of the BAC injection.

## Findings

### Methods: experiment 1

#### Animals

Ten naïve male and 10 naïve female Sprague-Dawley rats (Mean weight ± SEM = 440.15 ± 29.04 g; Mean age ± SEM = 129 ± 18 days), derived from the Harlan strain, were supplied by the Baldwin-Wallace College breeding colony (Berea, Ohio). Due to budget constraints the laboratory was only able to gain access to a certain number of rats from the institution's own breeding colony. The sex differences were of initial concern, but precautions were taken that included counterbalancing males and females within groups as well as ensuring animals' ages were consistent and animals' body weights were not statistically different between and within groups. Furthermore, analyses were run to check for differences in fluid consumption (SAC and water) on each day between sexes in each treatment group. Procedures were approved by the Baldwin-Wallace College Institutional Animal Care and Use Committee. Animals were procured and cared for according to the recommendations in the Guide for the Care and Use of Laboratory Animals [16] and in compliance with the Animal Welfare Act.

Throughout the experiment, the animals were housed in plastic 'shoe box' cages (20 cm × 22 cm × 20 cm deep) in a temperature-controlled room under a 12-hr light/dark cycle (lights on at 06:00 hrs). Rats had free access to food (Purina Rodent Chow, No. 5001, PMI Nutrition International, Brentwood, MO) but underwent fluid deprivation as described below. Throughout the study, the daily bottle weight differentials were recorded to the nearest 0.1 g (1 g of liquid = 1 ml of liquid).

#### Drug treatments and groups

Rats were randomly assigned to one of four drug-treatment groups (see Table 1). The groups received either 1 mg/kg (i.p.) of BAC (***BAC 1 mg/kg***; *N *= 5), 2 mg/kg (i.p.) of BAC (***BAC 2 mg/kg***; *N *= 5), or 3 mg/kg (i.p.) of BAC (***BAC 3 mg/kg***; *N *= 5). Male and female rats were randomly assigned to drug treatment groups. This resulted in 2 males and 3 females or vice versa in each group. All BAC injections were administered at a volume of 1 ml/kg. A fourth group of animals received sterile physiological saline (0.9% sodium chloride; 1 ml/kg, i.p.) (***SAL***; *N *= 5). All chemicals were purchased from Sigma Aldrich (St. Louis, MO) and drugs were mixed in sterile physiological saline immediately prior to injections. The baclofen used in this study was a racemic mixture: (±)-β-(Aminomethyl)-4- chlorobenzenepropanoic acid.

**Table 1:** Experiment 1 - Group nomenclature and treatments

Group Nomenclature	N	First Taste Exposure	Discrimination Tests Days 1 and 3	Rest Days Days 2 and 4
***SAL***	5	SAC^a^	SAL^b ^+ SAC^a^	Water

***BAC 1 mg/kg***	5	SAC	BAC^c ^+ SAC	Water

***BAC 2 mg/kg***	5	SAC	BAC + SAC	Water

***BAC 3 mg/kg***	5	SAC	BAC + SAC	Water

^a^SAC = two-bottle SAC preference test using 0.3%SAC and 0.6%SAC [%w/v; SAC salt dissolved in deionized water to specific concentration]

^b^SAL = physiological saline injection (0.9% NaCl dissolved in deionized water; 1 ml/kg, i.p.)

^c^BAC = BAC injection (BAC dissolved in physiological saline to either 1, 2 or 3 mg/ml; 1 ml/kg, i.p.; dosing specified in group nomenclature)

#### Procedures

Two days prior to the first tastant discrimination test, all animals were introduced to a 23-hr fluid deprivation cycle. The fluid deprivation cycle consisted of a 1 hr presentation of fluid/day to ensure animals were motivated to drink at the time liquid was present. On the first day of fluid deprivation, animals were given 1 hr of water only. The 1 hr fluid presentation occurred at the same time of day throughout the study (12:00-13:00 hrs).

On Day 1 of the study, rats were simultaneously offered 0.3% and 0.6% SAC solutions (2 bottle test) for 30 min (this is referred to in Table 1 as "First Taste Exposure"). The First Taste Exposure day was an attempt to reduce the effects of neophobia during the subsequent days when we used SAC consumption to assess BAC's ability to act as a US and its effects on taste discrimination. While rats will sample from both concentrations, they will reliably show a preference for 0.3% SAC over 0.6% SAC [refer to [17]]. However, water-deprived rats also tend to drink voraciously from the first source of liquid they encounter. Therefore in this study, the positions of our 2 bottles/cage were switched at 1, 5 and 10 min into the first 30-min presentation of liquid each day. Immediately following the 2-bottle SAC presentations the animals were given water for 30 min to prevent dehydration.

Discrimination testing took place on Days 2 and 4 of the study during which animals were given an injection of BAC or SAL 30 min prior to any SAC exposure. BAC's half-life is approximately 4 hrs following i.p. administration of the drug in rats and it has been shown to become pharmacologically active within 15 min, [2,18]. Thirty min following the injections animals were simultaneously presented with two different bottles of SAC (0.3% and 0.6%, %w/v) for a 30 min period, and bottle switching occurred as described for CS presentation on Day 1, the First Taste Exposure Day.

Animals were given one rest day (Day 3) following the first discrimination test during which they were allowed 1 hr access of water only and were given no drug injection. This rest day was designed to allow rehydration and time for the metabolism of the drugs given the day before. Animals were given two bottles of water during the first 30 min so that bottle positions could be switched as described above.

#### Statistical analysis

A Repeated Measures Analysis of Variance (RM-ANOVA) test was used to evaluate the differences in volumes of 0.3% and 0.6% SAC consumed by rats in each of the drug treatment groups on the discrimination test days as well as the first taste exposure day [SAC Concentration (0.3% or 0.6%) × Drug Treatment (SAL, 1, 2, 3 mg/kg BAC) × Test Days (first taste exposure, discrimination test 1 and test 2)]. We also ran one-way ANOVAs and Tukey post hocs to determine differences between drug-treatment groups for the following measures: the total SAC consumption (0.3% SAC + 0.6% SAC) per day, total fluid consumption (SAC + H20; on BAC-injection days 2 and 4) per day, and water consumption on Day 3 (when there were no injections and only water was offered). A one-way ANOVA and Tukey post hoc tests were run to determine if there was a significant difference in total fluid consumption or H_2_O consumption among treatment groups [Drug treatment: SAL, 1, 2, 3 mg/kg BAC]. The RM-ANOVA and one-way ANOVA with Tukey post hoc tests previously noted were repeated using 'milliliters per 100 g of body weight' (ml consumed/100 g) instead of the direct milliliter consumption measure in order to ensure there were no variances in reported observations that could be attributable to body weight differences. Furthermore, a one-way ANOVA was also run to determine if body weights were significantly different within or between groups. Finally, a *t*-test was run to compare daily fluid consumption (SAC and water) of males versus females in each group.

All statistical analyses were run using the Statistical Package for Social Sciences software (SPSS) (Chicago, IL) and α was set at 0.05 for all tests.

#### Results: experiment 1

**Figure 1:**
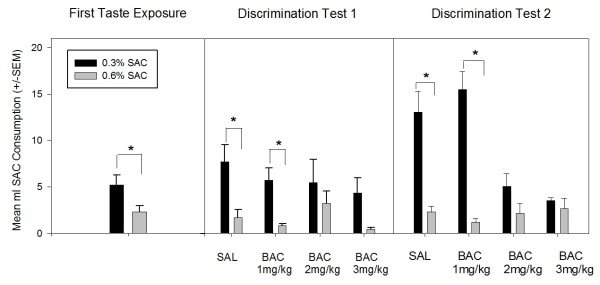
**Experiment 1-Evaluation of 0.3% versus 0.6% SAC Discrimination**. Mean ml SAC Consumption (± SEM) on the First Taste Exposure Day and both SAC Discrimination Test Days. RM-ANOVA showed that on the First Taste Exposure, in the absence of any drug treatment or other behavioral manipulation, all animals drank significantly more 0.3% SAC in comparison to 0.6% SAC. Also, RM-ANOVA showed that on both SAC Discrimination Test Days the SAL and BAC 1 mg/kg groups drank significantly more 0.3% SAC than 0.6% SAC. The BAC 2 mg/kg and BAC 3 mg/kg groups did not drink significantly different amounts of 0.3% SAC and 0.6% SAC on either test day. The SAL and BAC 1 mg/kg groups also showed a steady increase in 0.3% SAC consumption between the First Taste Exposure and Test 2 (the final taste exposure), but the BAC 2 mg/kg and BAC 3 mg/kg groups did not increase their consumption of 0.3% SAC. * Significant within group difference between 0.3% and 0.6% SAC. *p *< 0.05

On the first taste exposure day, when no drug injections were given, all groups showed a preference for 0.3% SAC despite the overall low SAC consumption due to neophobia [Mean SAC consumptions ± SEM: 0.3% SAC = 5.2 ± 1.1 ml; 0.6% SAC = 2.3 ± 0.7 ml]. Further, the ***SAL ***and ***BAC 1 mg/kg ***groups showed a statistically significant preference for 0.3% SAC over 0.6% SAC on both discrimination test days (when drug injections were administered prior to SAC presentation), indicating that their taste discrimination was intact. Saline-treated rats drank significantly more 0.3% SAC than 0.6% SAC during both discrimination tests as shown by RM-ANOVAs [Test 1: F(1, 3) = 10.260, *p *= 0.049; Test 2: F(1, 4) = 18.978, *p *= 0.022]. The ***BAC 1 mg/kg ***group also drank significantly more 0.3% SAC than 0.6% SAC on both test days [Test 1: F(1, 4) = 11.014, *p *= 0.029; Test 2: F(1, 4) = 49.239, *p *= 002]. However, the ***BAC 2 mg/kg ***and ***BAC 3 mg/kg ***groups drank statistically similar volumes of each SAC concentration on both test days, demonstrating a disruption in taste discrimination. Refer to Figure 1 for graphical representation of the first taste exposure day and the discrimination test days.

A significant drug treatment effect on total SAC consumption (0.3% + 0.6% SAC) and total fluid consumption (SAC + H2O) was observed on days when BAC was administered [F(3, 16) = 9.148, *p *= 0.001]. Total SAC consumption was significantly lower in the ***BAC 2 mg/kg ***and ***BAC 3 mg/kg ***groups compared to the ***SAL ***and ***BAC 1 mg/kg ***groups. Mean total fluid consumption on these BAC-injection days was significantly less for ***BAC 3 mg/kg ***animals compared to ***SAL ***and ***BAC 1 mg/kg ***animals [F(3, 16) = 6.717, *p *= 0.004], an indication of dose-dependent hypodipsia. However, the mean fluid consumption on BAC injection days did not differ significantly between the ***BAC 2 mg/kg ***animals compared to any of the other three groups. Total fluid consumption (H2O only) on the rest day (Day 3), when BAC was not administered, did not differ among any drug-treatment groups.

A dose-dependent effect on overall SAC consumption was demonstrated across the two discrimination test days [F(3, 15) = 7.059, *p *= 0.004]. There was a significant decrease in total SAC consumption from the first discrimination test day to the second discrimination test day in the ***BAC 2 mg/kg ***and ***BAC 3 mg/kg ***groups. Moreover, total SAC consumed was significantly less in these groups compared to the ***SAL ***and ***BAC 1 mg/kg ***group, as illustrated by Tukey *post hoc *comparisons. Additionally, the ***SAL ***and ***BAC 1 mg/kg ***groups showed a significant increase in total SAC consumption from the first taste exposure day to the second discrimination test day; this effect was opposite the decrease in SAC consumption observed in the rats receiving higher doses of BAC.

There were no significant differences between SAC or water consumption between males and females within the same group, as illustrated by *t*-test comparisons, nor was there a significant difference in average weight among drug-treatment groups, as illustrated by a one-way ANOVA comparison. Furthermore, the statistical differences reported in fluid consumption remained consistent when 'ml fluid consumption per 100 g body weight' was used as the dependent variable in the RM-ANOVAs and one-way ANOVAs, indicating that weight variances did not impact reported results.

### Discussion: experiment 1

Our first experiment showed that, in a dose-dependent manner, BAC appeared to disrupt drinking behavior in rats and limited their ability to discriminate between 2 different SAC concentrations. But, due to the significantly decreased SAC consumption from discrimination test 1 to test 2 observed in the ***BAC 2 mg/kg ***and ***BAC 3 mg/kg ***groups, it was still unclear as to what extent BAC was only altering the ability of rats to discriminate between the two different SAC concentrations and to what extent BAC may have either been exerting putative amnesiac effects or simultaneously acting as an US. Thus, we wished to further investigate the two BAC doses that induced disruptions in taste discrimination tests (***BAC 2 mg/kg ***and ***BAC 3 mg/kg ***groups).

In the following experiment animals were presented with a novel taste (0.3% SAC) paired with BAC or LiCl. Lithium chloride was chosen as a comparison measure since it is a more conventional and well-established US found in many other taste aversion studies [15,18-21]. If animals steadily decreased their SAC consumption to nearly zero over the three acquisition trials we could conclude that BAC was acting as an US. On the other hand, if animals continued to drink levels of SAC comparable to the first presentation of SAC (i.e. persistent neophobia), we could conclude that animals were experiencing anterograde amnesia due to BAC, an effect typical of GABA agonist administration [1,22].

### Methods: experiment 2

#### Animals

Twenty-nine naive male Sprague-Dawley rats (Mean weight ± SEM = 467.74 ± 38.43 g; Mean age ± SEM = 117 ± 10 days), derived from the Harlan strain, were supplied by the Baldwin-Wallace College breeding colony (Berea, Ohio). Rats had free access to food but underwent fluid deprivation as described below. Procedures were approved by the Baldwin-Wallace College Institutional Animal Care and Use Committee. Animals were procured and cared for according to the recommendations in the Guide for the Care and Use of Laboratory Animals [16] and in compliance with the Animal Welfare Act.

#### Drug treatments

Animals received one of three main drug treatments throughout the conditioning phase: LiCl (81 mg/kg; i.p.), BAC (2 mg/kg; i.p.) or BAC (3 mg/kg; i.p.) These doses of BAC were chosen based on the observations of Experiment 1 showing that they not only caused a disruption in SAC discrimination but also significantly reduced SAC consumption compared to the SAL or 1 mg/kg BAC injection groups. The 81 mg/kg dose of LiCl (i.p.) was chosen specifically based on our previous work demonstrating that 3 pairings of SAC and LiCl (81 mg/kg, i.p.) create a strong aversion to SAC in adult rats that is only extinguished upon multiple exposures of SAC alone [refer to [21]]. All drug solutions were made immediately prior to injections. Chemicals were purchased from Sigma Aldrich (St. Louis, MO). The baclofen used in this study was a racemic mixture: (±)-β-(Aminomethyl)-4- chlorobenzenepropanoic acid.

Three groups (***SAC + LiCl***, ***SAC + BAC 2 mg/kg***, and ***SAC + BAC 3 mg/kg***) received traditional taste aversion training in which the CS tastant was immediately followed by injection of the US (either 81 mg/kg LiCl, 2 mg/kg BAC, or 3 mg/kg BAC). In addition to the groups receiving conventional CTA training, there were three explicitly unpaired (EU) control groups [***EU(LiCl)***, ***EU(BAC 2 mg/kg) ***and ***EU(BAC 3 mg/kg)***] that received the CS tastant and 24 h later received a 30 min presentation of water followed immediately by an injection of either LiCl (81 mg/kg) or BAC (2 mg/kg or 3 mg/kg). The ***EU(LiCl) ***group revealed how normal, non-conditioned animals drink SAC, while simultaneously controlling for residual effects of LiCl and SAC exposure through the explicitly unpaired, non-associative method. Likewise, we controlled for the residual effects of BAC (2 and 3 mg/kg) in non-conditioned animals using the aforementioned ***EU(BAC 2 mg) ***and ***EU(BAC 3 mg) ***groups that received three explicitly unpaired exposures to BAC (2 or 3 mg/kg) and SAC. Refer to Table 2 for group nomenclature and treatments.

**Table 2:** Experiment 2 - Group nomenclature and treatments

Group Nomenclature	N	Conditioning		Extinction
			
		Days 1, 3, 5	Days 2, 4, 6	
***SAC + BAC 2 mg/kg***	5	SAC^1 ^+ BAC^2^	Water	SAC

***SAC + BAC 3 mg/kg***	4	SAC + BAC	Water	SAC

***SAC + LiCl***	5	SAC + LiCl^3^	Water	SAC

***EU(BAC 3 mg)***	5	SAC	Water + BAC	

***EU(BAC 2 mg)***	5	SAC	Water + BAC	

***EU(LiCl)***	5	SAC	Water + LiCl	

^1^SAC = 30 min presentation of 0.3% SAC [%w/v; SAC salt dissolved in deionized water]

^2^BAC = BAC injection (BAC dissolved in physiological saline to either 2 or 3 mg/ml; 1 ml/kg, i.p.; dosing specified in group nomenclature)

^3^LiCl = LiCl injection (81 mg/ml LiCl dissolved in physiological saline; dose = 81 mg/kg, i.p)

#### CTA acquisition

Animals were introduced to a 23-hr fluid deprivation schedule 2 days prior to the CTA conditioning phase of the study. Two 30-min presentations of water were given during these days, separated by 15 min (12:00-12:30 hrs and 12:45-13:15 hrs). This fluid deprivation paradigm has been used in previously published studies by our own laboratory [21]. Alternate and less severe fluid deprivation methods, including a gradual reduction in fluid presentation [23,24] were less appropriate for our study since it was imperative that all animals were similarly and highly motivated to drink upon the first CS exposure. Since the animals were given an hour access to fluid every day, the stress induced by this deprivation schedule should have been minimal. There is other literature supporting similar fluid deprivation schedules in conditioned taste aversion paradigms, which not only validate the procedure but also allow for easier comparison of our methods and findings [4,14,25-27].

The conditioning phase lasted a total of 6 days (see Table 2). On days 1, 3 and 5 of the study, all groups of animals were presented with SAC (0.3%;%w/v) for a 30 min period. Immediately following the drinking session animals were injected (i.p.) with one of the two doses of BAC or saline, depending on their group assignment. The ***SAC ***+ ***LiCl ***group was given an injection of LiCl (81 mg/kg, i.p.) at this time while the ***EU(LiCl), EU(BAC 2 mg)***, and ***EU(BAC 3 mg) ***controls received a physiological saline injection (1 ml/kg, i.p.) following the presentation of SAC. Fifteen min after the injections, these 3 groups were given another 30-min presentation of water to prevent dehydration. On the rest days (days 2, 4 and 6), the ***SAC ***+ ***LiCl ***and ***SAC ***+ ***BAC ***animals were not given any drug injections and were presented with water for two 30-min sessions, separated by 15 min.

The ***EU ***controls received both a CS (SAC) and US (LiCl, BAC 2 mg/kg, or BAC 3 mg/kg depending on designated group) presentation, but in an explicitly unpaired (EU) temporal relationship that prevented the formation of an association, but controlled for residual effects of repeated SAC exposure and the known, strong US properties of LiCl [28]. Animals in the ***EU ***groups received SAC (CS) on the conditioning days, just as the ***SAC + LiCl ***and ***SAC + BAC ***animals. On the following day (the rest days) the ***EU ***animals were given an injection of either LiCl or BAC (2 or 3 mg/kg), 24 h after the SAC presentation.

#### CTA extinction (EXT)

After the conditioning phase the animals were maintained on the 23-hr fluid deprivation schedule, but presented with SAC for 30 min daily. To prevent dehydration the animals were given an additional 30 min water-drinking session, 15 min after SAC exposure each day. The animals were maintained in this regimen until they reached asymptotic extinction (90% reacceptance of SAC as compared to baseline SAC drinking) [21]. Note: The ***EU ***control groups were not included in the extinction portion of the study since they were already drinking high levels of SAC at the end of the "conditioning" phase of the study and had no CTA to be extinguished.

#### Statistical analysis

A repeated-measures ANOVA [Drug Treatment (LiCl, 2, 3 mg/kg BAC) × Day (Conditioning day 1, 3, 5)] allowed us to analyze SAC consumption over the first three conditioning days (first 3 CS exposures). Subsequent Tukey post hoc tests were used to determine if there was a significant difference in SAC consumption among drug treatment groups during conditioning. The RM-ANOVA previously noted was repeated using 'milliliters per 100 g of body weight' (ml consumed/100 g) instead of the direct milliliter consumption measure in order to ensure there were no variances in reported observations that could be attributable to body weight differences. Furthermore, a one-way ANOVA was also run to determine if body weights were significantly different within or between groups.

Consistent with the criterion set by Nolan and colleagues [21,29], the end point for asymptotic extinction in our experiment was defined as SAC consumption greater than or equal to 90% of the baseline [9,21]. A one-way ANOVA and Tukey post hocs [Drug Treatment: LiCl, 2, 3 mg/kg BAC] were used to determine if there was a significant difference in the number of days to reach asymptotic EXT across drug treatment groups.

As a first step in evaluating the degree to which the rats in this study had extinguished their CTA, we needed to estimate levels of baseline familiar saccharin drinking. However, recording several days of baseline saccharin pre-exposure in our animals would have impeded future CTA training, due to latent inhibition effects [30]. Moreover, we also wanted to record saccharin consumption over several days to avoid the bias associated with the rat's initial hesitation to consume novel substances (neophobia) [31]. Therefore, baseline saccharin consumption was determined by averaging saccharin consumption on the third day of exposure from a separate group (*N *= 10) of similarly-sized rats maintained on the same fluid restriction schedule as the rats in the studies reported here (see CTA Acquisition section, above). This produced a mean saccharin consumption (± SEM) = 17.57 ± 1.29 ml [21]. In order to confirm that this method of determining baseline saccharin consumption was consistent with other ways to estimate familiar saccharin drinking, we also measured the saccharin consumption of a group of rats (*N *= 24; also maintained on the same fluid restriction schedule as the rats in the studies reported here) that were exposed to saccharin and LiCl but did not have the US and CS paired. Saccharin or LiCl were available/administered on alternate days. The saccharin consumption of this group represented normal enhanced acceptance of the sweet tasting liquid in the absence of conditioned avoidance. The animals that had these explicitly unpaired CS-US exposures over 3 saccharin-exposure days drank amounts of the sweet liquid (Mean + SEM = 18.2 + 2.8 ml) not significantly different from those animals that only drank saccharin over the same time period (see data above). In a final pilot study, we employed 7 fluid-restricted rats on the same 23-hr fluid deprivation schedule. Like the rats in the main study that went through CTA acquisition, these pilot animals were offered saccharin every-other day but, instead of receiving LiCl immediately after the saccharin, these rats received an equal volume of physiological saline (i.p.). On their third day of saccharin drinking, these rats drank 17.10 ± 1.38 ml (Mean + SEM) of the sweet liquid - an amount very similar to the baseline saccharin consumption estimates from the other methods described above. These data validated our method of estimating baseline saccharin consumption as a comparison point to determine 90% reacceptance of saccharin as asymptotic extinction.

### Results: experiment 2

**Figure 2:**
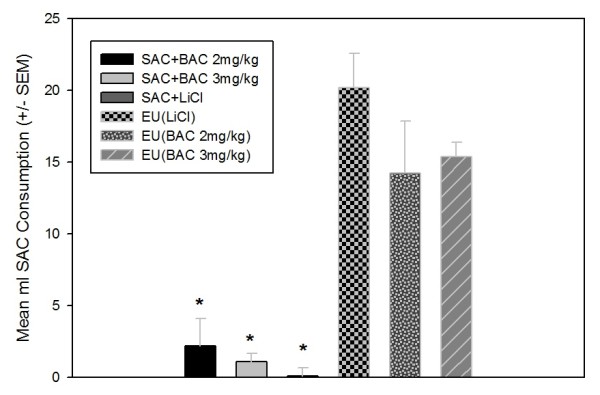
**Experiment 2-CTA Acquisition**. Mean ml SAC. Consumption (± SEM) on the Final Acquisition Trial. All animals that received either SAC + LiCl or SAC + BAC pairings acquired a strong taste aversion to SAC by the third CTA trial, as illustrated by the significant difference in SAC drinking between animals given EU training and those that had SAC paired with LiCl or BAC. All of the EU groups that did not receive CS + US pairings did not acquire a CTA, as indicated by high SAC drinking on trail 3, which was significantly greater than their drinking on trial 1. The SAC + LiCl, SAC + BAC 2 mg/kg and SAC + BAC 3 mg/kg groups were all drinking near-zero amounts of SAC on trial 3, which was significantly less than their SAC drinking on trial 1. Drinking on the first conditioning trial was not significantly different between any groups [Mean SAC consumption ± SEM on trial 1 = 4.35 ± 1.22 ml]. Note: A BAC 1 mg/kg group was not used in Experiment 2 because this group did not indicate a disruption of SAC discrimination capabilities in Experiment 1 nor did they demonstrate any possible US effects of BAC exposure in Experiment 1. *Significant difference between EU groups and the following CS + US groups: SAC + LiCl, SAC + BAC 2 mg/kg, and SAC + BAC 3 mg/kg. *p *< 0.05

All animals that received either a pairing of SAC + LiCl or SAC + BAC acquired a CTA by the third conditioning trial. A one-way ANOVA showed no significant differences between groups on the first SAC exposure day (trial 1). However, the ***EU(LiCl), (EU)BAC2 mg/kg ***and ***(EU)BAC3 mg/kg ***drank significantly more SAC on the third trial compared to the ***SAC + LiCl ***and both ***SAC + BAC ***groups, as illustrated in Figure 2 [F(5, 29) = 19.208, *p *< 0.001]. No other differences between these groups were observed on this final acquisition trial.

**Figure 3:**
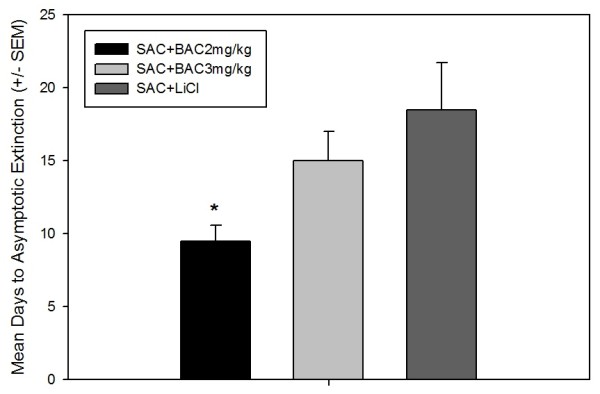
**Experiment 2-CS-Only Extinction**. Mean Days (± SEM) to Asymptotic EXT. The LiCl and BAC 3 mg/kg groups did not differ significantly in the number of days required to reach asymptotic EXT. However, the BAC 2 mg/kg group extinguished significantly faster than the LiCl and BAC 3 mg/kg groups. * Significantly less than both LiCl and BAC 3 mg/kg groups. *p *< 0.05

As illustrated in Figure 3, the number of days required for animals to reach asymptotic extinction of their CTA was not significantly different between the ***SAC + LiCl ***controls and the ***SAC + BAC 3 mg/kg ***rats. However, the rats treated with BAC 2 mg/kg did extinguish significantly faster than both the ***SAC ***+ ***LiCl ***and ***SAC + BAC 3 mg/kg ***animals [F(2, 11) = 5.902, *p *≤ 0.018]. ***EU ***controls (receiving either LiCl or BAC explicitly unpaired with SAC) were not included in Figure 3 because they were drinking "asymptotic levels" of SAC on EXT day 1 and had no aversion to be extinguished.

There were no significant differences in average weights among drug-treatment groups, as illustrated by a one-way ANOVA comparison, nor were there significant within-group differences. Furthermore, the statistical differences reported in fluid consumption remained consistent when 'ml fluid consumption per 100 g body weight' was used as the dependent variable in the RM-ANOVA for conditioning, indicating that weight variances did not impact reported results.

## Discussion

Experiment 1 revealed that BAC dose-dependently (2 and 3 mg/kg, i.p.) reduced 0.3% vs. 0.6% SAC discrimination capabilities as well as total fluid consumption in rats. Moreover, within-group comparisons indicated that 1 mg/kg BAC did not alter gustatory discrimination or consummatory behaviors. At the higher doses of BAC, we observed a decrease in liquid consumption (both SAC and water) that is consistent with symptoms of gastro-intestinal upset and/or neophobia [7,9]. While neophobia would explain the decreased drinking of SAC and water on the first BAC injection day, it would not explain the subsequent decrease within ***BAC 2 mg/kg ***and ***BAC 3 mg/kg ***groups on the second BAC injection day, in experiment 1. In previous rat studies, we have observed that neophobia to SAC disappears quickly, as unconditioned animals increase their drinking from the first to the second taste exposure and by the third taste exposure animals are drinking asymptotic amounts of the SAC [refer to [21]].

Both doses of BAC tested in experiment 2 (2 and 3 mg/kg) did induce a CTA, as SAC consumption decreased significantly over 3 CS + US pairings in all animals receiving SAC + BAC. This suppression was comparable to that observed in rats that received SAC + LiCl. Therefore, while compatible with some previous findings that BAC does affect rats' solid and liquid consummatory behaviors [10,11,14], our experiment 2 additionally indicated that induction of visceral malaise, by BAC (2 and 3 mg/kg) may lie behind certain behavior-modulating effects of baclofen on liquid consumption. However, our observations that a CTA formed as a result of pairing SAC + BAC over 3 consecutive trials may be contrasted with observations of Chester & Cunningham [13]. Chester and Cunningham [13] reported that 2 mg/kg (i.p.) BAC did not show US (malaise-causing) properties or significantly affect SAC consumption, in control animals, after one SAC + BAC pairing. Also, in their mouse study, BAC (2 mg/kg, i.p) injections took place immediately after SAC exposure. This may be compared to the timing in both of our experiments (either 30 min prior to SAC exposure, as in experiment 1, or paired immediately after SAC exposure as the US, as in experiment 2). The number and timing of CS-US presentations and faster metabolism of mice compared to rats may partially explain this difference in the assessment of BAC's US properties in the context of a CTA paradigm [2,12]. Additionally, as in our experiment 1, the decreased consumption after one pairing of SAC followed by BAC 2 mg/kg may not have reached statistical significance. However, the results in our experiment 1 did indicate that consumption patterns between ***BAC 2 mg/kg ***animals were different from SAL control animals in that consumption of SAC did not increase from the first to second SAC exposure.

An investigation into the differences in SAC consumption patterns between the ***SAL ***and ***BAC 1 mg/kg ***groups compared to the ***BAC 2 mg/kg ***and ***BAC 3 mg/kg ***groups determined that BAC (2 and 3 mg/kg) can be used as a reliable US in CTA experiments. Experiment 1 indicated that BAC at 1 mg/kg exhibited no US properties in rats. However, all rats that received BAC 2 or 3 mg/kg or LiCl paired with SAC over three trials exhibited a strong CTA in Experiment 2. We found that BAC (3 mg/kg) induced a conditioned taste aversion to SAC that extinguished in a time-course similar to that of a more conventional LiCl-induced SAC aversion [21]. However, the animals that received ***SAC + BAC(2 mg/kg) ***pairings took less time to extinguish their CTA than did rats receiving ***SAC + BAC(3 mg/kg) ***or ***SAC + LiCl(81 mg/kg)***. The "floor effect" observed during acquisition may have obscured an indication of the varying intensities of the CTA in each group, a difference which was then only later revealed during EXT.

One may argue that the ***SAC + BAC(2 mg/kg) ***animals were not tasting the SAC on Day 3 or EXT Day 1 while the other groups may have been tasting more, on average, so the ***SAC + BAC(2 mg/kg) ***group had only 2 effective pairings of SAC + BAC. But, average SAC consumption in all groups did not differ significantly and all were consuming near-zero SAC. It was observed that most animals were at least tasting the SAC (consumption ≤ 0.4 ml), so the argument that they may have been getting the US in absence of the CS is not applicable in this situation. Even so, as there were a few animals that drank zero SAC on the final acquisition day, the time of SAC presentation was controlled in all groups. Barnfield and Clifton [32], showed that the duration of time the CS is present is actually a more potent indicator and control for taste aversion than is the volume of CS consumed. This further supports a claim that the difference in malaise-inducing properties of the US injections were dose-dependent, rather than dependent on any variability in final CS consumption. The dose-dependency of BAC's visceral effects, furthermore, would be a plausible explanation for the observed differences in extinction times; BAC at 2 mg/kg is a weaker US, while BAC at 3 mg/kg is comparable to the malaise-inducing properties of LiCl (81 mg/kg, i.p.).

Due to the cascade of neurophysiological effects BAC produces in the CNS [9,33] and its observed toxicity at very high doses, it is not surprising that BAC may have been perceived as noxious at doses used in our experiments. Through direct action as a GABA_B _agonist, BAC indirectly reduces levels of a variety of other neurotransmitters (e.g., norepinephrine, dopamine, acetylcholine, serotonin, glutamate, aspartate and GABA) [2,6,9,34]. Such neurotransmitter changes are capable of inducing nausea, dizziness or confusion, among other effects, and may very well be physiological mediators of CTA formation in BAC-treated animals (at doses of BAC above 1 mg/kg) [1,2,33]. Therefore, BAC may possess US properties through the modulation of these various neurotransmitter systems. Furthermore, Inui and colleagues [35] showed data indicating that blockade of GABA_A _receptors in the ventral pallidum is capable of altering the taste palatability of SAC in a CTA paradigm. In our second experiment, the **EU(BAC 2 mg/kg) **and **EU(BAC 3 mg/kg) **rats did not consume significantly different amounts of SAC compared to the **EU(LiCl) **animals, indicating that there was likely not a significant SAC palatability shift. However, it is important to note such effects and realize that there could be a variety of explanations and mechanisms driving the consummatory behaviors in ours and other experiments, due to the wide ranging effects of the GABAergic system. In the present studies there appears to be a dose dependency of BAC on consummatory behaviors: at 2 mg BAC, and even more so at 3 mg/kg BAC, the US effects of BAC are manifested in an observable manner via measurable and statistically significant changes in the animals' consummatory behaviors and taste discrimination capacity. At 1 mg/kg (i.p.), the behavioral effects associated with BAC's toxicity or sensorium-altering capabilities appear negligible for taste discrimination and aversion investigations.

While it is clear that BAC (2 and 3 mg/kg) altered the animals' sensorium and was successfully used to induce an observable CTA to SAC (indicated by a stark decrease in SAC consumption following SAC + BAC pairings that was only restored after repeated SAC-only presentations) the mechanism by which BAC exerts these US effects is still unclear. While CTAs are indeed induced by malaise-inducing agents (such as LiCl) and animals' natural survival mechanisms that drive them to avoid tastes associated with illness [22], this is not always the case. While there is literature to support that an aversion may not be formed in absence of gastro-intestinal distress [refer to [36-38]], there is also new literature indicating that drugs inducing any change in an animal's sensorium, altering their psychological or physical state negatively or positively, can also induce a CTA to a novel tastant. For example, Parker [34,39] showed that animals can exhibit taste avoidance (which may be interpretted as an aversion) when CS tastants are paired with drugs possessing rewarding effects. Goudie and colleagues [40] also showed that conditioned nausea was not always the necessary mediator of drug-induced conditioned taste aversions. However, while avoidance to a novel tastant may be induced by both positively and negatively reinforcing drugs, a conditioned taste aversion induced by malaise inducing (negative reinforcing) properties will be coupled with the Lay-On-Belly (LOB) response [20] and conditioned disgust reactions such as chin rubs and gapes [18]. Although we did not record these conditioned disgust reactions, an argument that BAC may have induced malaise is strengthened by the observation that animals receiving SAC + BAC pairings continued to avoid the SAC into the EXT phase of the study even though they were not receiving any BAC injection during this phase. Future studies, however, should look at the conditioned disgust reactions in attempt to elucidate whether or not the taste avoidance and observed aversion in the present study was due to malaise-inducing, emetic, or other negative reinforcing properties of BAC or possibly due to another mechanism related to BAC's effects on other neurotransmitter levels.

Overall, our data indicate that BAC given at 2 or 3 mg/kg (i.p.) impairs sensory abilities and decreases gustatory discrimination in rats. But, these effects may be augmented by dose-dependent US properties of BAC that, when paired with the neutral gustatory stimulus, SAC, induced a CTA in Experiment 2. Additionally, in Experiment 2, rats acquired a strong CTA over 3 SAC + BAC pairings and only over multiple days of CS-only EXT training did SAC consumption return to asymptotic levels. These data carry implications for future experiments that seek to use BAC in the context of consummatory paradigms. While studies available in the most current literature avoid confounds of BAC administration (for examples refer to 12, 23, 34), our study highlights the importance for investigators to control for the dose-dependent US properties induced by systemic BAC injections. The side-effects of BAC at doses equal to or greater than 2 mg/kg (i.p.), in rats, can inadvertently alter results and affect conclusions drawn from taste dependent paradigms. However, BAC may still successfully be used in future CTA manipulations at doses equal to and less than 1 mg/kg (i.p.), since our behavioral measures did not differ between saline control animals and those receiving the lowest dose of BAC in Experiment 1. Development of novel GABA_B _agonists void of taste-altering or malaise-inducing effects would benefit future studies aimed at determining the role of GABAergic neurons in taste aversion learning.

## Conclusions

The GABA_B _agonist, BAC, decreased the ability of rats to differentiate between 0.3% and 0.6% saccharin in a two bottle preference test, when the drug was administered intraperitoneally (i.p.) at doses of 2 mg/kg or 3 mg/kg. At 1 mg/kg (i.p.), BAC showed no signs of disrupting gustatory discrimination, nor signs of inducing a taste aversion to SAC. However, at 2 mg/kg and 3 mg/kg, BAC showed unconditioned stimulus effects that were sufficient to create a CTA to SAC. Futhermore, 3 mg/kg BAC induced a CTA to SAC that was comparable to the more conventional SAC + LiCl (81 mg/kg) paradigm. Future experiments should include observations of orofacial responses indicative of conditioned disgust (e.g., gapes, chin rubs, paw treads) and avoidance. Subsequent studies may also include more extensive observations of visceral malaise such as the Lay-On-Belly measure in order to fully conclude that the conditioned taste aversion and avoidance induced by BAC in the present study was attributable to gastrointestinal distress associated with the BAC injection instead of other central aversive effects that may have also led in part to the observed hypodipsia in lieu of visceral malaise.

## List of Abbreviations

(BAC): Baclofen; (CTA): Conditioned taste aversion; (CS): Conditioned stimulus; (US): Unconditioned stimulus; (EU): Explicitly unpaired; (EXT): Extinction; (GABA): Gamma-aminobutyric acid; (i.p.): Intraperitoneal; (p.o.): Per oral; (LiCl): Lithium chloride; (NMDA):N-methyl-D-aspartate; (SAC): Saccharin

## Competing interests

The authors declare that they have no competing interests.

## Authors' contributions

All authors contributed in the drafting and submission of the experimental protocol to the Institutional Animal Care and Use Committee, as well as carrying out all experimental procedures and data collection. All also took part in statistical analyses and interpretation of results as well as the writing of the research paper. All authors have read and approved the final manuscript.
